# VCGIDB: A Database and Web Resource for the Genomic Islands from *Vibrio cholerae*

**DOI:** 10.3390/pathogens8040261

**Published:** 2019-11-23

**Authors:** YoungJae Hur, Mauricio Chalita, Sung-min Ha, Inwoo Baek, Jongsik Chun

**Affiliations:** 1 Interdisciplinary Program in Bioinformatics, Seoul National University, Seoul 08826, Korea; yjhur82@snu.ac.kr (Y.H.); mauricio@snu.ac.kr (M.C.); 2 ChunLab Inc., Seoul 06725, Korea; smha118@snu.ac.kr; 3 Institute of Molecular Biology and Genetics, Seoul National University, Seoul 08826, Korea; beoptim@snu.ac.kr; 4 School of Biological Sciences, Seoul National University, Seoul 08826, Korea

**Keywords:** comparative genomics, database, genomic island, *Vibrio cholerae*

## Abstract

*Vibrio cholerae* is the causative agent of cholera, which is a severe, life-threatening diarrheal disease. The current seventh pandemic has not been eradicated and the outbreak is still ongoing around the world. The evolution of the pandemic-causing strain has been greatly influenced by lateral gene transfer, and the mechanisms of acquisition of pathogenicity in *V. cholerae* are mainly involved with genomic islands (GIs). Thus, detecting GIs and their comprehensive information is necessary to understand the continuing resurgence and newly emerging pathogenic *V. cholerae* strains. In this study, 798 *V. cholerae* strains were tested using the GI-Scanner algorithm, which was developed to detect candidate GIs and identify them in a comparative genomics approach. The algorithm predicted 435 highly possible genomic islands, and we built a database, called *Vibrio cholerae* Genomic Island Database (VCGIDB). This database shows advanced results that were acquired from a large genome set using phylogeny-based predictions. Moreover, VCGIDB is a highly expendable database that does not require intensive computation, which enables us to update it with a greater number of genomes using a novel genomic island prediction method. The VCGIDB website allows the user to browse the data and presents the results in a visual manner.

## 1. Introduction

*Vibrio cholerae*, the causative agent of cholera, remains a global public health threat. The disease causes severe and acute diarrhea, which is potentially followed by shock, acidosis, and death [1]. There have been seven pandemics of cholera since 1817, with the seventh pandemic beginning in 1961 in Makassar, Sulawesi, Indonesia. *V. cholerae* strains are classified into serogroups based on the somatic O antigen, and there are over 200 serogroups [2] of *V. cholerae*. Before the emergence of an epidemic caused by O139 across Bangladesh and India in 1992, serogroup O1 was the only serogroup that had caused outbreaks [3]. The seventh pandemic of cholera was caused by O1 strains of the El Tor biotype, whereas the sixth pandemic was caused by O1 strains of the classical biotype. The evolution of the seventh-pandemic strains has been greatly influenced by lateral gene transfer (LGT) [4].

Genomic islands (GIs) are clusters of genes that are mobile via mechanisms of lateral gene transfer. GIs are known as an essential mechanism in the acquisition of many novel phenotypes, such as pathogenicity and antibiotic resistance [5]. Crucially important GIs such as VPI-1, VPI-2, VSP-1, and VSP-2 are very well studied in comparative genomics and phylogenetic analysis [4,6]. In a previously published work [6], it was shown that a single clade of strains with a common ancestor received several GIs (VSP-1, VSP-2, GI-11) and were diversified by several significant lateral gene transfers that occurred in their natural environment, causing the current pandemic. Moreover, the multidrug-resistant strains of *V. cholerae* are reported to be caused by the acquisition of the SXT integrative conjugative element [7]. Thus, there is a clear need for the detection of lateral gene transfer and a comprehensive GI database for *V. cholerae*.

There are few numbers of genomic island databases, including IslandViewer4 [8], PAIDB [9], and Pre_GI [10]. The IslandViewer database provides prediction results of multiple methods without clustering predicted GIs, but separated results from each genome. PAIDB is a very focused database that specifically provides pathogenic islands detected from prokaryotic genomes. Pre_GI identifies GIs and groups similar genomic islands. Those databases are an excellent tool for GI research, but the coverage of analyzed genome data still remains very low in numbers.

Due to the technological improvement in genome sequencing, the number of whole sequenced genomes has been increasing rapidly during the past few years, which has resulted in a large number of genomes that come from phylogenetically close strains or the same species. Also, most computational methods are limited by the number of genomic sequences, some sets are too small to predict GIs using comparative genomics. However, for *V. cholera*, there is a need for an algorithm that is able to handle a large set of genomic sequences in order to do comparative genomics in an efficient manner.

Here, we created *Vibrio cholerae* Genomic Island Database (VCGIDB) by comparing the genome sequences of 798 *V. cholerae* strains, which is a vast set of single-species genomes for GI prediction. To predict these GIs, we created an algorithm, named GI-Scanner, which uses a large set of genomes and its phylogeny in order to detect and identify GIs. The predicted results from GI-Scanner were then stored in the VCGIDB website, which also allowed for the results to be browsed and visualized. In this study, we validated VCGIDB by comparing other GI databases and previous comparative genomics algorithms. Finally, we introduce VCGIDB and its corresponding website (http://leb.snu.ac.kr/vcgidb).

## 2. Results

From 798 *V. cholerae* strains, GI-Scanner algorithm predicted 435 highly possible genomic islands, including 67 genomic islands already found in a previous study [6]. The average genome size of *V. cholerae* used in this study was 4,022,915 bp with 3582 ORFs. Only five or more continuous ORFs among the predicted result were identified as GIs. Annotations for GIs were indexed in VCGIDB, which contained designated GI names from previous studies, the existence of antimicrobial resistance (AMR) genes, and the possible mobile functionalities. Genomic islands that shared more than 80% of ORFs, but were mismatched in their neighboring ORFs, were categorized as the same genomic island with a separated GI-cluster. Twelve GIs had multiple clusters, which implied that those GIs were inserted into multiple sites within their genomes. GI-Scanner predicted three groups of accumulative genome sets, which shows a gradual expansion of the prediction results. The summary of these sets and their number of prediction results are described in Table 1. GI-Scanner also detected 983 candidate GIs that are considered to have occurred by a deletion event or a repeat of copied genes on several genomes. Those negative data were also stored in VCGIDB together in order to avoid redundant calculation in future analysis.

Antibiotic resistance genes were also searched and annotated by the Resistance Gene Identifier (RGI) program from the CARD database [11], confirming six dominant multi-drug-resistant genes embedded in SXT elements: *floR*, *strA*, *strB*, *sulII*, *tetR*, and *tetA*. Additionally, another multi-drug-resistant gene containing a genomic island was also identified. This genomic island, VCGI-131, inserted between VC0768 and VC0769, carried a similar array of multi-drug-resistant genes in SXT elements: *sulII*, *strA*, *strB*, and *tetR,* respectively. No other SXT element genes were found hovering over the antibiotic resistance gene cluster. Only eight strains carried the antibiotic resistance gene cluster among the 21 strains inserted VCGI-131. (2012Env-92, 254-93, CISM_300205, FORC_073, HE-40, HE-46, HE39, HE48).

**Figure 1:**
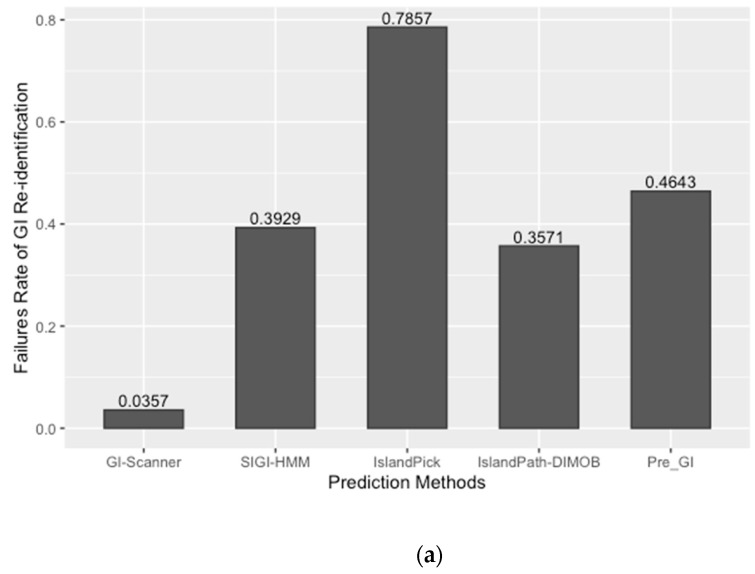

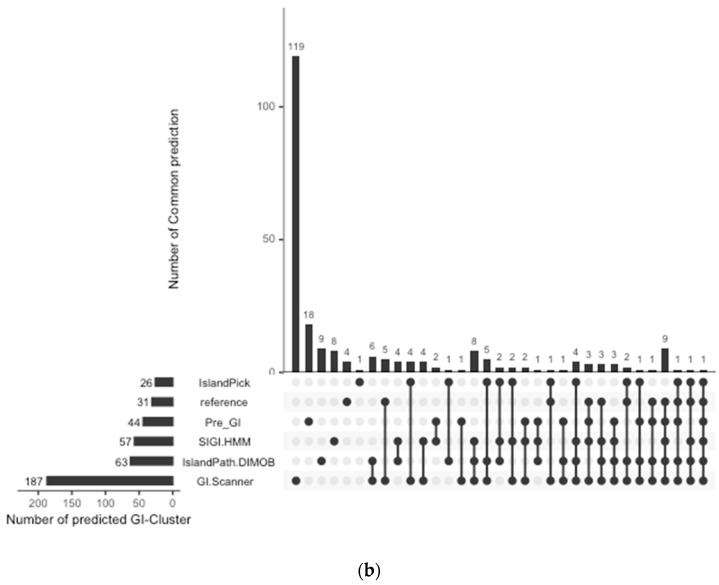
Comparison of GI prediction results from different databases or methods. (**a**) Re-identification failures rate for GI databases: False-negative rates were calculated by failures rate of re-identification of 28 reference GIs from previous study [6]. The strains that were not included in all databases were excluded during the comparison. (**b**) Prediction results overlapping with other GI databases: All prediction results were collected and clustered in the same genomic context. The GI-Scanner algorithm outperformed due to the massive genome comparison.

The prediction result was compared with previously published work and other GI databases for validation and calculation of false negative rates (FNR). In this study, 67 reference GIs for *V. cholerae* were collected from previous studies, excluding GIs from low-quality genomes [6]. The FNR was defined as the percentage of the failures to re-identify 28 reference GIs from the genome set that all genomic island databases commonly analyzed [10]. The Calculated FNR is described in Figure 1a. GI-Scanner predictions had a lower FNR of 3.57%, while others showed relatively high FNR: 39.29% with SIGI-HMM [12], 78.57% with IslandPick [13], 35.71% with IslandPath-DIMOB [14], and 46.43% with Pre_GI [10]. Furthermore, using 67 reference GIs, GI-Scanner showed 5.88% FNR, and only four GIs were missed; two consisted of less than five ORFs, one covered by multiple contigs, and only one that was never detected. For a more detailed comparison, GI prediction results among the databases can be seen in Figure 1. By excluding strains that were not included in other databases, we overlapped the predictions across six other prediction methods and plotted them. The GI-Scanner algorithm predicted 187 GIs, out of which 119 GIs cannot be found in other databases. The four GIs that GI-Scanner missed were never predicted by other prediction methods.

**Figure 2:**
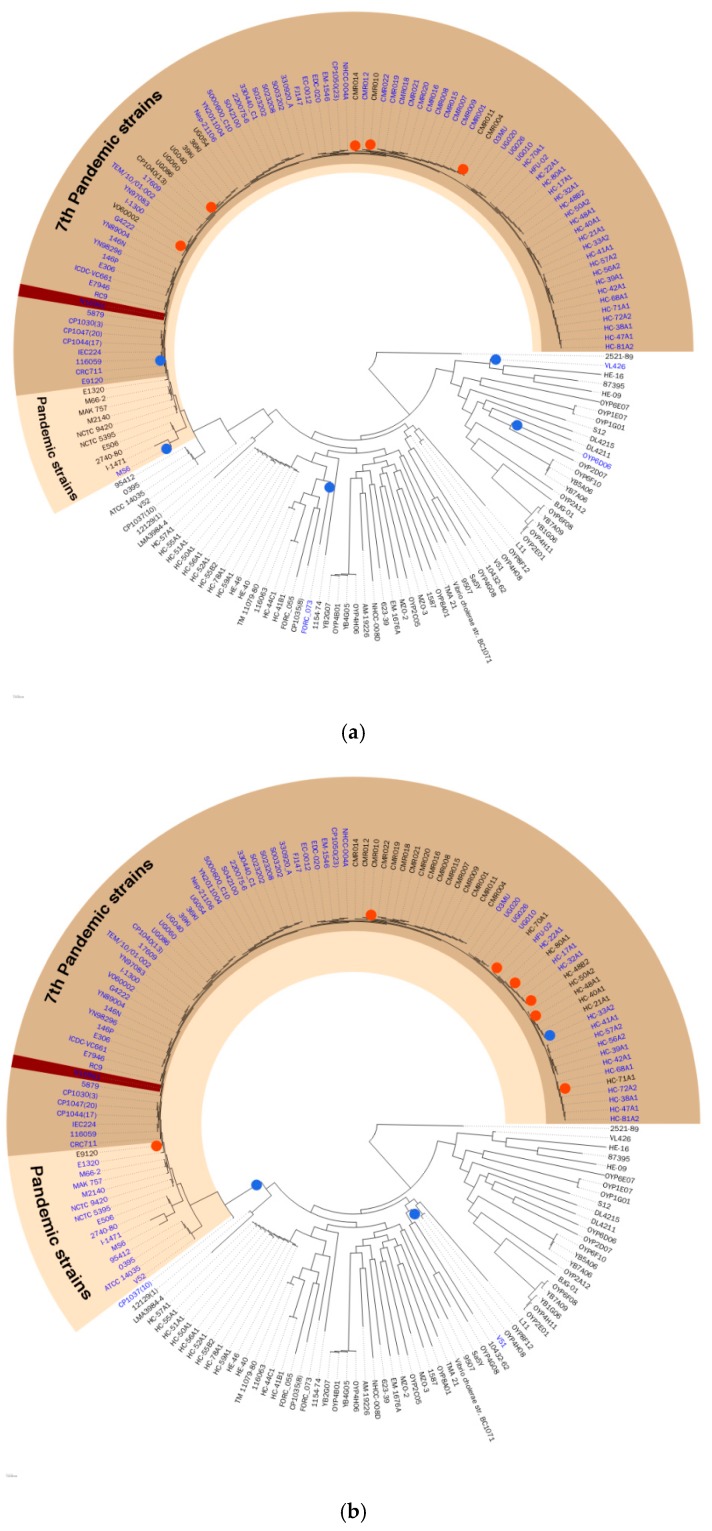
The hypothetical evolutionary history of the (**a**) VPI-1 and (**b**) VSP-1 genomic islands. The 171 selected strains were shown on the phylogeny tree. The pandemic strains and the seventh pandemic strains were shaded with colors. Blue dots were marked where the hypothetical insertion event occurred, and red dots were marked where the hypothetical deletion event occurred. GI containing strain were labeled with blue. The reference strain N16961 was marked with dark brown highlight.

A website was developed for the visualization and browsing of VCGIDB. The website provided a list of GIs and a list of genomes from *V. Cholerae*, which contained detailed information of a specific GI within any genome. The novelty of this work is that aside from information of a single GI, it presents the same GIs in the same genome context from large genome sets of *V. Cholerae*. VCGIDB also provides hypothetical evolution history of GIs shown as a phylogenetic tree (Figure 2), and pairwise genome comparisons tables enable users to see possible evidence of horizontal gene transfer. In addition, for each detailed GI page, we show visualized representative GI ORFs, similar GIs in the database, and notable ORFs, such as mobility-related genes or antimicrobial resistance genes. Comprehensive genome browsing provides a circular genome map that includes the annotation of the position of the GIs on that genome and provides a GC ratio graph with the baseline of 49.08%, which came from the GC ratio of 1440 single-copy core gene composite genomes. The website is accessible at http://leb.snu.ac.kr/vcgidb.

## 3. Discussion

In this study, using a large genome set for *V. cholerae*, we demonstrated that GI-Scanner was able to predict a high number of GIs that no other databases were able to predict. Most of the 119 GIs were found from genomes that were not used in different methods. It is a remarkable advanced result that could be acquired from a large genome set with close distance. The false-positive rate of GI-Scanner was never calculated in this study due to the lack of a reliable validation method to prove prediction results [10,15]. Thus, there can be an argument about the possibility that those novel GIs are false positives. However, results of genome comparisons and phylogeny tree comparison with the existence of GI-clusters are strong evidence of the existence of these GIs. It is worth noting those novel GI predictions, in spite of the limitation of a computational prediction method with the absence of complete GI data.

Figure 2 shows the hypothetical evolutionary history of each GI proposed in this study, VPI-1, and VSP-1, which was constructed by the projection of the binary existence of GIs onto the phylogeny tree. Pathogenic island VPI-1 was mostly found in pandemic strains, and its hypothetical insertion event happened recently from a common ancestor node of all pandemic strains. This hypothetical evolutionary history supports the differentiation of the *V. cholerae* pandemic strain occurring in VPI-1. Similarly, VSP-1, the seventh-pandemic *Vibrio*, island-1 has a hypothetical evolutionary history that supports differentiation of seventh-pandemic strains occurring by itself. This hypothesis is supported by other previous studies [4,16]. Hypothetical evolutionary history constructed by maximum parsimony excluded predicted GIs without a hypothetical insertion event. Thus, only GIs corrected with the possible evidence of horizontal gene transfer, which is the hypothetical insertion event, were presented in VCGIDB. The feature of a proposed hypothetical evolutionary history will give a comprehensive evolutionary understanding of GIs to researchers.

In general, comparative genomics approaches for GI prediction use multiple genome alignment algorithms such as MAUVE [13,17]. Expanding the test genome set required exponential time due to the absence of known result application. GI-Scanner accomplishes genome comparison and expands the GI-cluster pairwise, in a separated process that can be parallelized to reduce calculation time. In the future, VCGIDB will update rapidly increasing, newly sequenced *V.* *cholerae* genomes with novel GI predictions.

It is worth discussing how the simple observation from the result of prediction with the three groups of accumulative genome sets shows gradual expansion. Not surprisingly, the observation in Table 1 confirmed the fact that the completeness of assembly affected the result of the prediction. Prediction results from the first highly assembled strain group, to which 58 strains belong, show that only a small set of highly assembled genomes are enough to discover more than 45% of the entire prediction. This is because the longer contigs have more contextual information to apply comparative genomics methods. Thus, securing the number of the highly assembled genomes is still a limitation of the comparative genomics approach to detecting GIs.

## 4. Materials and Methods 

### 4.1. Genomes and Phylogeny of V. Cholerae

A total of 798 whole genome sequences belonging to *V. cholerae* were downloaded from NCBI Assembly Database (https://www.ncbi.nlm.nih.gov/assembly) after filtering out genomes with low quality, contamination, and redundancy as described earlier [18]. To generate a phylogenetic tree from a 798-whole-genome test set, we used a Single Nucleotide Variant (SNV)-based search algorithm. First, we generated a composite genome associated from 1440 single-copy genes found from 32 representative complete genomes using the Roary v3.12.0 [19] pipeline. The composite genome followed the sequence from the N16961 strain. This artificial composite genome was used as a reference for a pairwise SNV analysis, which was performed by MUMmer v3.23 [20] against all 798 test genomes. Based on the calculated SNVs, a maximum likelihood tree was obtained with RAxML [21] using the GTRCAT model option. Tree manipulation and visualization were implemented with an ETE3 [22] python plugin. The script ran with a maximum parsimony algorithm [23] to calculate minimum edit coast of existence status based on phylogeny tree and predicted gene cluster insertion or deletion with visualization.

### 4.2. Detecting Candidate GIs with GI-Scanner 

In this study, we developed GI-Scanner, an algorithm to detect and identify candidate GIs using comparative genomics. The algorithm compares composite genomes in a pairwise manner to map subject homologous ORFs to a given query. Homolog ORFs were found using the USEARCH [24] program in both directions of the composite genome with criteria of a bit-score ratio higher than 0.9. Non-aligned ORFs were clustered as a five-or-more continuous array of ORFs. Neighboring ORFs, five ORFs both forward and backward of those clusters, were tested to see if they were continuously mapped against another genome to detect the insertion region. The reliable clusters with a certain insertion region were collected for binning along with other clusters that came from further pairwise genome comparisons. The binning process used the frequency of the ORFs belonging to those secure clusters. The regions that had the ORFs with a more than four times higher frequency in comparison to their neighbors were selected as the candidate GI.

### 4.3. Identification and Annotation of Genomic Islands

A composite genome of a candidate GI and its neighboring ORFs were searched among the whole test set using GI-Scanner. Strains without a homologous neighboring region, defined as “unknown existence”, were excluded from the phylogenic analysis. Resulting composite genome sets were shared a specific genomic context of themselves as well as neighboring ORFs. We defined such a composite genome set as GI-cluster, which prevented having the same GI with a different insertion region. All GI-clusters were tested and determined for correction of GI by comparing phylogenetic trees and full binary existence matrices.

### 4.4. Annotation

All genes from 798 *V. cholerae* strains were functionally annotated by mapping those protein sequences to the top hit of the EggNog ver. 4.5 database [25] using the USEARCH program with e-value < 10^−5^. Functional annotation terms were subsequently filtered computationally with regular expressions, searching terms related to function of mobility; for example, transposon, plasmid, phage, carbohydrate-active, and other horizontal gene transfer (HGT) mechanisms [26]. Antibiotic resistance genes were detected by RGI. If genes were predicted as “PERFECT” or “STRICT” by the RGI program, the curated reference sequence and its antimicrobial resistance (AMR) model was annotated.

### 4.5. Testing the Expansion Capability of the Database

GI-Scanner was designed to automatically detecting either known or new GIs with a given set of genomes. Initially, a given genome was checked to see if it was already included in VCGIDB by GI-Scanner. The genome that had not been previously analyzed was then examined using GI-clusters in VCGIDB. GI-Scanner searched for known GI-clusters from the input genome and expanded the GI-cluster if the same GI existed. Those known GI-cluster regions were then masked out and only novel candidate GIs were detected from the input genome. To describe the ability of the database expansion, strains of *V. cholerae* were grouped into three according to their assembly status: 58 strains are classified as Complete and Chromosome, 219 strains as Scaffold, and the remaining are classified as contig. Three groups were analyzed in order, accumulatively.

### 4.6. Database Comparison and Validation

The result of GI-Scanner was compared with other genomic island databases and their prediction results, such as Pre_GI and IslandViewer, comprising IslandPick, SIGI-HMM, and IslandPath-DIMOB. In a previous study [6], 98 GIs were predicted with a similar comparative genomics approach to the one used in this study. Among the results from the previous work, 31 GIs were excluded due to genome quality issues or short ORF numbers, but 67 GIs were still validated and compared with other results. Because other databases did not provide GI-cluster information, the prediction results from all databases were collected and clustered with pairwise partial genome comparison, the same as with GI-Scanner clustering. GIs from strains that were not included in other databases or predicted were excluded during the comparison.

## 5. Conclusions

VCGIDB was successfully developed to present the results of GI predictions by GI-Scanner, and to provide comprehensive information, including functional annotations for proteins, ARM information, projection onto a phylogeny tree, the same GIs on other genomes, pairwise comparative genomics matrix, and the same GIs with different insertion sites. This database allows researchers to understand the diversity of GIs among a large number of strains within the species, using a comparative genomics analysis without tremendous computation.

## Figures and Tables

**Table 1:** Three groups of genome sets accumulated and expanded prediction results. Note that genomes from a higher assembly level predicted relatively more genomic islands (GIs).

Assembly Level	Accumulated Number of Strains	Accumulated Number of Predicted GIs
Complete or Chromosome	58	198
Scaffold	277	312
Contig	798	435
